# Supplementary material for: Crossed laser phase plates for transmission electron microscopy
Source: ArXiv. 2024 Oct 29:arXiv:2410.11328v2. Preprint. [Version 2] (PMC11527100)
Supplement: Supplement 1 [file NIHPP2410.11328v2-supplement-1.pdf]

Supplementary Note 1: Derivation of the relativistic XLPP phase shift

In the non-relativistic limit, the interaction between the electromagnetic field and the electron beam is given by the ponderomotive potential, which does not depend on the polarization of the electromagnetic field and repels the electrons from the high-intensity antinodes of the laser standing wave. For relativistic electron beams, as found in practice, the interaction becomes polarization-dependent. For electrons moving at velocities v_e larger than $c/\sqrt{2}$ (electron energies larger than $\sim$211 keV), the interaction can even become reversed, such that the antinodes appear attractive to the electron beam while the nodes become repulsive [11]. In this section, we will derive the relativistic phase shift profile created by the XLPP.

In the laboratory frame in Coulomb gauge, the phase shift η imparted to the electron matter waves by the laser is [11]

$$\eta = \frac{\mathrm{e}^2}{2m\hbar}\int \frac{dt}{\gamma}\left[\left(\mathbf{A}(\mathbf{d}_0(t),t) - \nabla G(\mathbf{d}_0(t),t)\right)^2 - \beta^2\left(A_z(\mathbf{d}_0(t),t) - \nabla_z G(\mathbf{d}_0(t),t)\right)^2\right], \tag{S1}$$

with $-\mathrm{e}, m$ the electron charge and mass, $\mathbf{A}$ the vector potential of the laser field with $A_z := \mathbf{A}\cdot\hat{\mathbf{r}}_z$, $\mathbf{d}_0(t)$ the unperturbed electron trajectory, G a gauge function that arises from the Lorentz transformation between the lab frame and the frame in which the uperturbed electron beam is at rest, $\beta = v_e/c$ the normalized electron velocity, and $\gamma = 1/\sqrt{1-\beta^2}$. The vector potential $\mathbf{A}(\mathbf{r},t) = \mathbf{A}_1(\mathbf{r},t) + \mathbf{A}_2(\mathbf{r},t)$ of the XLPP is given by adding two standing waves propagating in the $\hat{\mathbf{r}}_x$- and $\hat{\mathbf{r}}_y$-direction, respectively:

$$\mathbf{A}_1(\mathbf{r},t) = A_1(y,z)\cos(kx)\left[\cos(\theta_1)\cos(\omega t)\hat{\mathbf{r}}_z + \sin(\theta_1)\cos(\omega t - \varepsilon_1)\hat{\mathbf{r}}_y\right], \tag{S2}$$

$$\mathbf{A}_2(\mathbf{r},t) = A_2(x,z)\cos(ky)\left[\cos(\theta_2)\cos(\omega t + \Omega)\hat{\mathbf{r}}_z + \sin(\theta_2)\cos(\omega t - \varepsilon_2 + \Omega)\hat{\mathbf{r}}_x\right] \tag{S3}$$

where $\mathbf{r} := (r_x, r_y, r_z)$ are the spatial coordinates, θ_j are polarization angles relative to the $\hat{\mathbf{r}}_z$ axis, ε_j are ellipticity parameters, $k = 2\pi/\lambda_l$ is the angular wave number, and Ω is the temporal phase of the two standing waves. Assuming the envelope functions $A_{1,2}$ are slowly-varying relative to the wave cycle along the electron trajectory [11], we can time-average the integrand of Equation (S1) over one period $T = 2\pi/\omega$ of the optical field, writing η in terms of an effective potential U,

$$\eta = \frac{1}{\hbar}\int dt\, U(\mathbf{r}), \tag{S4}$$

$$U(\mathbf{r}) = \frac{\mathrm{e}^2}{2m\gamma}\frac{1}{T}\int_0^T dt\,\left[\left(\mathbf{A}(\mathbf{r},t) - \nabla G(\mathbf{r},t)\right)^2 - \beta^2\left(A_z(\mathbf{r},t) - \nabla_z G(\mathbf{r},t)\right)^2\right], \tag{S5}$$

where we note that $r_z = c\beta t$. In the slowly-varying envelope approximation, we can also approximate the gauge function as [11]

$$G(\mathbf{r},t) \approx \frac{c\beta}{\omega}\left[A_1(r_y,r_z)\cos(kr_x)\cos(\theta_1)\sin(\omega t) + A_2(r_x,r_z)\cos(kr_y)\cos(\theta_2)\sin(\omega t + \Omega)\right]. \tag{S6}$$

Under these conditions, Equation (S4) gives the phase shift for an arbitrary configuration of the parameters $\{\theta_1, \theta_2, \varepsilon_1, \varepsilon_2, \Omega\}$.

Special cases

We will consider the three special cases of laser beams polarized vertically, horizontally, and at the "relativistic reversal angle."

Vertical polarization. When the two laser polarizations are vertical ($\theta_{1,2} = 0$), we find

$$U(\mathbf{r}) = U_v(\mathbf{r}) = \frac{\mathrm{e}^2}{4m\gamma}\left\{\frac{A_1^2(r_y,r_z)}{2}\left[1 + (1-2\beta^2)\cos(2kr_x)\right] + \frac{A_2^2(r_x,r_z)}{2}\left[1 + (1-2\beta^2)\cos(2kr_y)\right] + 2A_1(r_y,r_z)A_2(r_x,r_z)\cos(kr_x)\cos(kr_y)\cos(\Omega)(1-\beta^2)\right\}. \tag{S7}$$

We note that the terms in A_j^2 have the same form as those in a single phase plate [11], wherein the effective potential appears as a standing wave with its modulation depth scaled by $(1 - 2\beta^2)$. In the non-relativistic limit $\beta \to 0$, we observe that the potential reduces to

$$U_v(\mathbf{r}) = \frac{\mathrm{e}^2}{4m\gamma}|A_1(r_y, r_z)\cos(kr_x) + A_2(r_x, r_z)\cos(kr_y)e^{i\Omega}|^2, \tag{S8}$$

such that the phase shift η is proportional to the integral of the laser intensity along $\hat{\mathbf{r}}_z$. However, we note that at the accelerating voltages used in cryo-EM, the β-dependent terms cannot be neglected. The consequences of this for the CTF are explored in Supplementary Note 2.

Horizontal polarization. When both laser polarizations are horizontal ($\theta_{1,2} = \frac{\pi}{2}$), the two laser beams can no longer interfere and the effective potential is simply the sum of that coming from two SLPPs,

$$U(\mathbf{r}) = U_h(\mathbf{r}) = \frac{\mathrm{e}^2}{4m\gamma}\left\{\frac{A_1^2(r_y, r_z)}{2}\left[1 + \cos(2kr_x)\right] + \frac{A_2^2(r_x, r_z)}{2}\left[1 + \cos(2kr_y)\right]\right\}. \tag{S9}$$

We can see that the resulting potential is similar to that of Equation (S7) in the limit $\beta \to 1$ except that the modulation depth of the standing waves is inverted in the latter case.

Relativistic reversal angle polarization. When $\beta \geq 1/\sqrt{2}$, the polarization-dependence of the laser-electron interaction permits a unique feature of η. At the so-called relativistic reversal angle (RRA), $\theta_r := \arccos(1/\sqrt{2}\beta)$, the nodes and antinodes of a laser standing wave create the same phase shift [11]. In this case, the potential of the XLPP simplifies to

$$U(\mathbf{r}) = U_r(\mathbf{r}) = \frac{\mathrm{e}^2}{4m\gamma}\left\{\frac{1}{2}\left[A_1(r_y, r_z)^2 + A_2(r_x, r_z)^2\right] + \frac{1-\beta^2}{\beta^2}A_1(r_y, r_z)A_2(r_x, r_z)\cos(\Omega)\cos(kx)\cos(ky)\right\}. \tag{S10}$$

The phase shift away from the origin takes on a smooth gaussian shape, while near the origin the pattern is complicated by the interference of the two laser beams. The phase pattern that results is plotted in Figure S1.

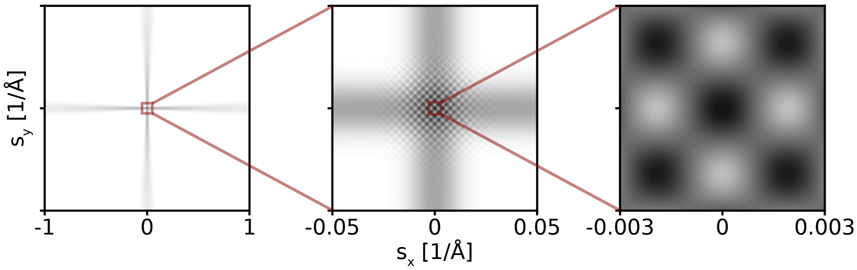

Figure S1: **Phase shift at the relativistic reversal angle.** Progressively zoomed-in view of the phase shift produced by a XLPP with both laser beams polarized at the RRA (with $N_A = 0.08$). Middle panel shows the difference between the central region where the beams interfere and the outer regions where the standing wave structure of the laser intensities is washed out by the polarization-dependent relativistic effect. White corresponds to zero phase shift and black to the maximum.

Supplementary Note 2: Effects of laser polarization

As shown in Supplementary Note 1, the phase shift η produced by the XLPP depends significantly on the polarization of the laser beams. In this section, we consider the effects of laser polarization on the CTF by examining the three special cases derived above: when the lasers are polarized vertically, horizontally, and at the RRA.

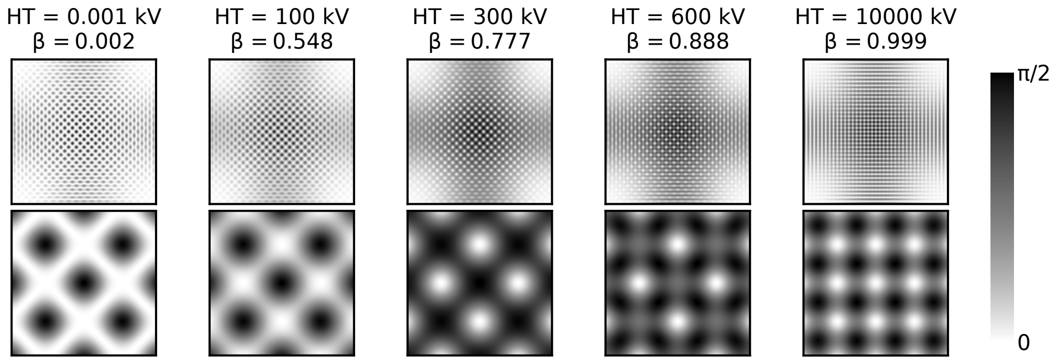

Figure S2: **Dependence of phase shift η on electron velocity β in a vertically-polarized XLPP.** Each column shows the phase shift η as experienced by electrons with a different accelerating voltage (HT) through a XLPP with vertically-polarized ($\theta_{1,2} = 0$) lasers. Bottom panel is zoomed in 10× around the center relative to the top panel and in all cases the phase shift is linearly scaled so that the peak phase shift is $\frac{\pi}{2}$ (black).

Vertical polarization maximizes the interference of the two lasers. The cross-term produced by laser interference results in a favorable cut-on frequency in the non-relativistic case (Equation S8), but examination of the relativistic interaction reveals that this feature gradually disappears and the cut-on frequency gradually increases as the electron velocity β is increased (Figure S2). It should be noted that λ_e changes with β, which affects the size of the diffraction pattern.

When the lasers are horizontally-polarized, they cannot interfere, so the cut-on frequency remains fixed as β is varied. For low β, therefore, this configuration produces a worse cut-on frequency than vertical polarization, but as seen in Figure S2, the performance of the latter is limited at high β. The transition occurs around an accelerating voltage of ~100 kV, as shown in Figure S3.

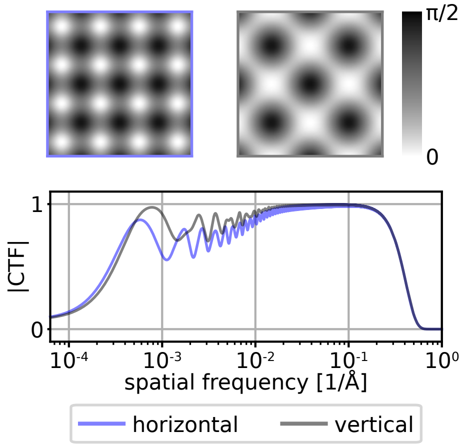

Figure S3: **Polarization-dependence of η at 100 kV accelerating voltage.** Top panel shows the zoomed-in phase shift for horizontally-polarized beams (left) and vertically-polarized beams (right). In both cases, laser power is set so that peak phase shift is $\frac{\pi}{2}$. Bottom panel shows the azimuthally-averaged modulus of the CTF.

When the accelerating voltage exceeds ~211 kV, operation of the lasers at the RRA is a viable option to eliminate the modulation of η along the laser beams at high spatial frequencies, as seen in Figure S1. Relative to horizontal polarization, the ghost image contrast is suppressed by ~20 % in this configuration, and the smoothing of η at high spatial frequencies suppresses the high-spatial-frequency components in the ghost images (Figure S4). However, the cut-on frequency is somewhat worse.

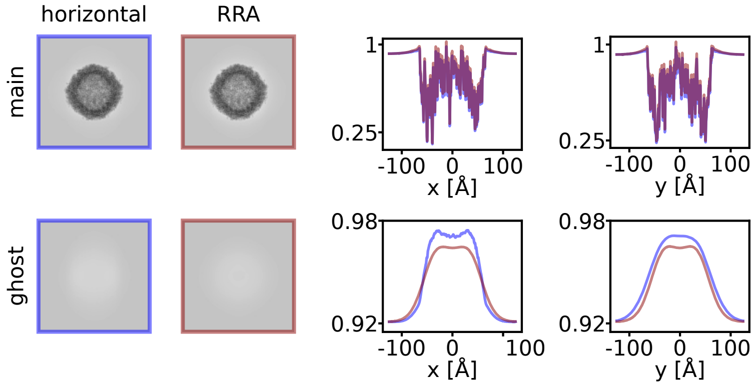

Figure S4: **Ghost suppression by relativistic reversal.** Top row shows a main image of an isolated apoferritin particle for a XLPP with $N_A = 0.08$ and either horizontal (blue) or RRA (red) polarization. Horizontal (x) and vertical (y) line scans through the centers of the two images are shown. Bottom row shows the same plots for first-order ghost images. RRA polarization suppresses peak ghost contrast by $\sim$20 %. Horizontal scans show that high-spatial-frequency information in the ghost is lower with RRA polarization than horizontal polarization.

Evidently, vertical polarization is favored at accelerating voltages at or below $\sim$100 kV for its low cut-on frequency, while horizontal polarization is favorable at higher voltages. At voltages above $\sim$211 kV, operation at the RRA may be desirable for its suppression of ghosts. However, the reduction in ghost contrast is modest and the amplitude of high-spatial-frequency components of ghosts is relatively low (Figure 3), so it remains to be seen whether this configuration is worth the considerably more demanding alignment than the horizontal XLPP. Figure S5 compares the azimuthally-averaged modulus of the CTF between horizontal and RRA polarizations of the XLPP at the very high N_A of 0.2, demonstrating that, despite a slight increase in cut-on frequency, the latter yields contrast transfer satisfyingly close to the ideal Zernike phase plate.

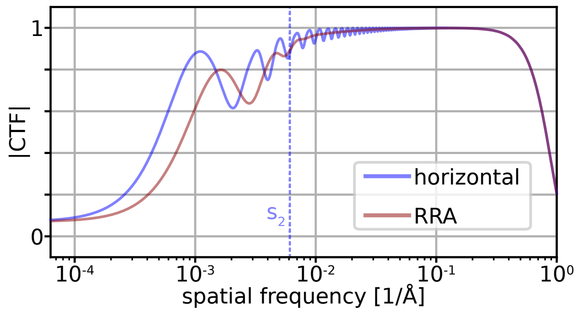

Figure S5: **Contrast transfer at N_A of 0.2.** Azimuthally-averaged modulus of the CTF is plotted for horizontal and RRA polarizations of the XLPP. The former has a lower first cut-on frequency but the latter does not have amplitude oscillations above s_2 due to the washing out of the standing wave patterns away from the laser intersection.

Supplementary Note 3: Removal of CTF oscillations

As discussed above, the use of a phase plate in cryo-EM obviates the need for defocus and spherical aberration. Operating with a SLPP or XLPP in the "in-focus" condition with spherical aberration correction ($Z = C_s = 0$) yields the CTFs shown in Figure 1d, which have no oscillations at high spatial frequencies. This results in a doubling of the spectral power of images at the high spatial frequencies relative to the usual case of $Z \approx -1\,\mu\text{m}$ and $C_s \approx 2.7\,\text{mm}$. For comparison, the latter configuration is illustrated in Figure S6, both with and without a LPP. While incorporating a phase plate into this configuration increases the CTF substantially at low spatial frequencies ($|\mathbf{s}| < s_2$), the phase plate alone is not sufficient to eliminate the oscillations of

the CTF at high spatial frequencies, which are caused by the terms in χ (Equation (6)) which are quadratic (defocus) and quartic (spherical aberration) in $|\mathbf{s}|$.

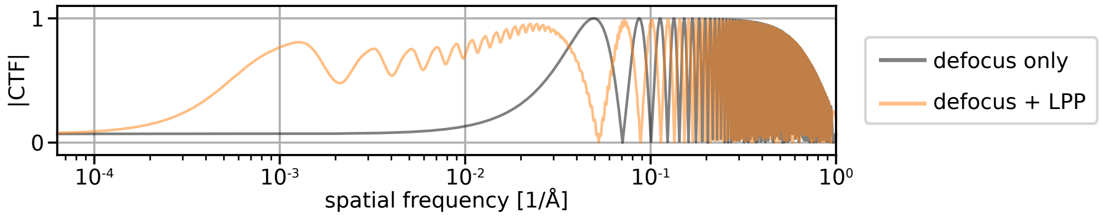

Figure S6: **CTF oscillations caused by defocus and spherical aberration.** Azimuthally-averaged modulus of the CTF is plotted for the case of $Z = -1\,\mu\text{m}$ and $C_s = 2.7\,\text{mm}$ in the absence of a phase plate (gray) and when using a SLPP with $N_A = 0.05$ (orange). Additional calculation parameters are provided in Table S1.

Supplementary Note 4: Calculation parameters

Table S1 lists the typical values of relevant parameters used in the calculations throughout this study. Deviations from these values are specified in the text, figure captions, and/or figure legends.

Parameter	Symbol	Value
focal length	f	14.1 mm
energy spread (FWHM)	–	0.3 eV
amplitude contrast	κ	0.07
specimen thickness	–	250 Å
chromatic aberration	C_c	5.1 mm
spherical aberration	C_s	2.7 mm or zero
defocus	Z	$-1\,\mu\text{m}$ or zero
electron wavelength	λ_e	1.97 pm
mirror radius of curavture	R	10 mm
laser wavelength	λ_l	1064 nm
laser polarization ellipticities	$\varepsilon_{1,2}$	zero
laser polarization angles	$\theta_{1,2}$	$\pi/2$ rad
laser temporal phase	Ω	zero

Table S1: Values of parameters used in calculations throughout the paper, unless otherwise indicated. Parameters indicated with – are not assigned a symbol in the text. The non-zero values of Z, C_s are used in the "conventional" configuration in Figure S6; elsewhere, the "in-focus" condition is used, with $Z = C_s = 0$.
